# The Importance of a More Thorough Knowledge of the Dental Organs on the Part of Physicians, to the Successful Management of Many Diseases—and Equally Requisite, a More Thorough Knowledge of the General System by Dentists, for Greater Skill and Success in the Practice of Their Art

**Published:** 1856-07

**Authors:** W. R. Handy


### ARTICLE VI.

*The Importance of a more thorough Knowledge of the Dental Organs on the part of Physicians, to the successful management of many Diseases—and equally requisite, a more thorough knowledge of the General System by Dentists, for greater skill and success in the practice of their art.* By W. R. HANDY, M. D.

IN the July No. of this journal for 1855, the first branch of the above subject was considered—we now invite attention to the second branch. To the first the attention of physicians was more particularly called, and the charge made of slighting the *dental organs*, in their anatomical studies, in comparison with the other organs of the body, and, as a necessary consequence, failure in the management of many diseases. To the second branch—which is the subject now before us—we ask the especial attention of the dentist, particularly the practicing dentist and dental student.

Dentists and students of dentistry are charged with the *want* of a more thorough knowledge of the *general system*, and, as a necessary consequence, failure in the acquisition of that skill and success so necessary and desirable in the practice of their art.

The importance of such thorough knowledge of the general system by the dentist, we are aware, is not generally acknowledged as necessary in the practice of his profession; and the acquisition of such knowledge, therefore, is considered a waste of time which might be more profitably spent in other matters. Regarding this point of great practical moment, we may be pardoned for appearing to dwell at some length upon it. This question involves the knowledge necessary to make a safe and skillful dentist on the one hand, and the health, happiness, and lives of suffering patients on the other.

The latter have no security without the attainment of this necessary knowledge by the former, and the former, without such attainment, cannot practice safely and honestly without injury to the latter.

What, then, is this necessary knowledge to make a competent dentist? The answer has been stated to be a more thorough knowledge of all the organs composing the human body.

The oculist and the aurist each practices his specialty—the one upon the eye, the other upon the ear; each confining his treatment to the one organ, and both alike declining to treat the body generally. Does it, therefore, follow that these physicians have no knowledge of any other organs except the eye and the ear, to which they devote most of their time, and upon which they exercise most of their skill? On the contrary, precisely the reverse of this seems to be the truth in the case, and that the physicians who practice these specialties of the eye and the ear are generally among the most thoroughly educated in every department of the healing art.

They acknowledge the necessity, and unhesitatingly proclaim it, that a knowledge of all the organs is absolutely necessary to a safe and trustworthy practice upon either the eye or the ear, and their professional brethren sustain them just because they have studied all the organs in all their relations, and are thereby best qualified to practice on any individual organ, and without such qualifications would not be considered as entitled to fellowship with the scientific physicians of any age or any land. But further, the people at large seem to think it not only necessary, but to require that a physician practicing in either of the specialties of the eye and the ear shall also be as thoroughly qualified as he who treats the body generally. But for the dentist who practices upon the teeth, there seems to be an exception in his favor. He appears to be a much more privileged character than either the eye doctor or the ear doctor. It is not considered very necessary, generally speaking, for him to know any thing about the body except the teeth, and even about them not much seems to be required. The dentist, therefore, in the judgment of the great body of the medical profession, as well as in that of the people at large, appears to be quite exempt from the extended qualifications thought so requisite in the oculist and the aurist. The dental profession is scarcely regarded as having any rights in common with the medical, and, therefore, as having little claim to equality of fellowship in the great family of physicians.

Now, we ask, why this difference—this great difference between the dentist, who practices upon the teeth as his favorite specialty, and that of the oculist and the aurist, who practice upon the eye and the ear as their favorite specialties? Why should the qualifications of the former be considered less necessary than those of the latter? And why should there be the less credit or the less dignity in the preservation of the teeth, than in that of either the eye or the ear? These questions bring us more directly to our subject, which is to examine the statement, i. e. that a more thorough knowledge of the general system by dentists, is requisite for greater skill and success in the practice of their art.

The first reason we have to assign why a general knowledge of all the organs of the body is as necessary to the dentist as to the physician who treats exclusively either the eye or the ear, is, that he is thus put in possession of all the resources of the body, whether for weal or woe, which may bear upon and influence the teeth, just as the physician who obtains a like knowledge of the resources and influences which the body brings to bear upon the eye and the ear. Each alike, dentist as well as physician, can turn the knowledge of such influences to a practical advantage, and in a twofold degree, first, by benefiting himself in greatly extending his skill and success; and secondly, by benefiting his patients in greatly adding to their health and happiness.

This reason is subordinate to one still more fundamental and conclusive on this point. We allude to the unity of the body and dependency of all its various parts.

That each organ is dependent, to a greater or less degree, on every other organ composing the human body, each upon the whole and the whole upon each, is, we believe, an acknowledged principle, and not called in question by any one.

Every organ, therefore, however inferior in function and position it may seem, is consequently related with every other organ, and exerts, according to the amount of its relationship, more or less influence upon each and all the organs. This being admitted, it appears to necessarily follow, that the teeth, as parts and organs of the body, must come in for their share of influence with each and all the other organs of said body. Granting such influence to exist, it must as necessarily follow that this influence is reciprocal, and that the relation of the teeth with the rest of the body, and of the body with the teeth, is as mutual and necessary for the preservation of health, as the acknowledged and necessary relationships of either the eye or the ear. If this be true, how can we avoid the conclusion, that it is not as necessary for the dentist to have qualifications as extended for successful practice upon the teeth, as that of the oculist and aurist for like success upon the eye and the ear? And as a still further consequence, the absolute necessity of dentists, equally with physicians, making the whole body their study, that they may thus be the better prepared to practice any particular specialty? An objection here arises, and at one period was urged with some degree of plausibility, i. e. that the teeth, though admitted to be organs, nevertheless occupied so low a place in the rest of the organism, that their vitality was so small, and influence so insignificant, and that they had no circulation except what was seen in the pulse. We say, with such notions as these, it is not difficult to understand why so little importance should have been attached to the teeth, and why the qualifications of a dentist should be regarded of such little consequence.

But now, that this day is past, and we all know that the teeth are organs, that they are vascular, and that they are in full fellowship with all the other organs of the body —we say, knowing this, can there be any excuse why dentists should not, as well as physicians, properly qualify themselves for the duties they have assumed?

As there still, however, seems to be the belief, to a very considerable extent at least, that the qualifications of a dentist need not by any means be so extensive as those of a physician, we propose to try to prove that this is an error, and an error which, in our humble judgment, we think, greatly retards the progress of dental improvement as well as detracts from the dignity of the dental profession.

We here, in advance, take pleasure in acknowledging the many illustrious names, dead and living, that have adorned, and struggled to bring dentistry to its present eminence. We also cheerfully express our satisfaction and approbation of the dental colleges and dental societies that have every where sprung up in our midst, to elevate the standard of dental science and forward the march of dental progress. All of this seems clearly to show the necessity of more extended dental qualifications than have hitherto existed, and all such movements we hail as the harbinger of still brighter and better days to come.

To hasten, if possible, this result, is our chief aim at present; which brings us back to the error stated above, in which the belief is inculcated that dental qualifications are not very necessary, or at least only so to a very limited extent.

The first remark we have to offer is, that the health of the whole body involves the health of its several parts, or that general hygiene includes special hygiene and of course must include the teeth. This would seem to necessarily follow from the admitted fundamental position of the unity and dependency of all the various organs of the body.

It may not be amiss, however, to illustrate this point, in its bearing more especially upon the teeth.

Nutrition, it is well known, is one of the great leading functions of the body—to the whole and every part of the body. It consists of several subordinate functions, as digestion, absorption, respiration, circulation, secretion, and assimilation; and it is the result of the combined agency of these subordinate functions which constitutes nutrition. Its chief design is also well known to be the nourishment of the body; and among the organs for preparing this nourishment the teeth occupy a very important place, for they are found in the very front ranks of the process, are among the first to begin digestion, and are regarded as indispensable in the preparation of this universal pabulum for all the organs.

This general nourishment or pabulum, known as the blood, is distributed in due proportion to every part, so that the teeth must come in for their share. Now it will be observed that the teeth are employed, along with other organs, in manufacturing this blood, which is afterwards brought back to nourish and sustain these same teeth in the discharge of their important function. Here we see a reciprocation of duties between the teeth and the blood—a relation natural, close, and inseparable—and further, a relation so inseparable with health, that if you destroy it, you not only destroy the teeth and the blood, but likewise bring a proportionate ruin to every other part.

The importance and necessity, yes, we may add, the absolute necessity, of every dentist studying this relation between the teeth and the blood, seem self-evident; and not simply of studying it superficially and isolated, but thoroughly and connectedly; when it will be seen that this relation is not confined to the teeth and blood, but extends to and is felt in greater or less degree in every part. And this is true whether examined in a state of health or disease. Hence it follows, that before we can at all claim to know well even this relation, it is also necessary we should study every other relation with which it is associated. This can be very readily understood, when we but for a moment reflect that the *blood* is the grand reservoir of all the materials for all the organs, and that each organ, however great or however small it may be, is absolutely dependent upon this fluid blood for its very existence, development, growth, and future maintenance. The teeth form no exception to this universal law.

Now, if the blood be in a healthy or physiological condition, we expect to find all the organs in the same state of health; but on the other hand, if the blood be diseased we must expect the organs to be diseased likewise. It may not be amiss to ask here, what constitutes a healthy condition of the blood? as the answer will greatly help us in determining the extent of qualifications necessary to make a competent dentist.

Mr. Paget in his recent lectures on surgical pathology says, that among the many conditions necessary to normal nutrition, there are four which are the chief and most prominent, i. e.—

"1. A right state and composition of the blood or other nutritive material.

2. A regular and not far distant supply of such blood.

3. A certain influence of the nervous system.

4. A natural state of the part to be maintained."

Upon the right state of the blood he thus remarks: "I may observe that I use the expression right state rather than purity, because if the latter be used, it seems to imply that there is some standard of composition to which all blood might be referred, and the attainment of which is essential to health ; whereas the truth seems rather to be, that from birth onwards the blood and tissues of each creature are adapted to one another, and to the necessary external circumstances of life ; and that the maintenance of health depends on the maintenance and continual re-adjustment of the peculiarities on which this exact adaptation depends." And he continues : "The necessity for this right or appropriate state of the blood, as a condition of healthy nutrition, involves of course the necessity for the due performance of the blood-making and blood-purifying functions; it requires healthy digestion, healthy respiration, healthy excretion. Any one of these being disturbed, the formative process in a part or in the whole body may be faulty for want of the appropriate material."

"But important as these are," he adds, "we must not let the consideration of them lead us to forget that there is something in the blood itself which is at least as essential to the continuance of its right and healthy state as these are, and which is, indeed, often occupied in correcting the errors to which these, more than itself, are subject ; I mean the power of assimilation or maintenance which the blood possesses, in and for itself, as perfectly and at least as independently as any of the tissues."

And he further adds, that by this power of assimilation it is "that notwithstanding the diversity of materials put into the blood, and the diversity of conditions in which the functions ministering to its formation are discharged, yet the blood throughout life retains, in each person, certain characters as peculiar as those of his outer features, for the continual renewal of which it provides appropriate materials. And by this assimilative power of the blood it is that the tissues are continually guarded ; for by it many noxious substances introduced into the blood are changed and made harmless before they come to the tissues ; nor can any substance, introduced from without, produce disease in an organ, unless it be such an one as can escape the assimilative and excretory power of the blood itself."

Symmetrical diseases, and next to these homologous diseases, are cited by Mr. Paget as proofs of the nicety, constancy, and perfect exactness of the adaptation between the blood and all the tissues, and by which adaptation the health of all is preserved.

In the language of this author it is the "perfect and most minute exactness of the adaptation which, in health, exists between the blood and all the tissues; and that certain inconceivably slight disturbances of this adaptation may be sources of disease."

Again, from the writings of Treviranus, our author takes the following quotation, i. e. "that each single part of the body, in respect of its nutrition, stands to the whole body in the relation of an excreted substance," and makes this comment upon it: "If each part," he says, "in its normal nutrition is as an excreting organ to the rest, then the cessation or perversion of nutrition in one must, by no vague sympathy, but through definite change in the condition of the blood, affect the nutrition of the rest, and be thus the source of constitutional disturbance."

From these quotations we wish to make a remark as showing their practical bearing upon the teeth, and the consequent practical qualifications of the dentist, necessary in view of such relation. This practical relation is equally strong and direct with every other organ, but as we are most concerned, at present, with the relation of the blood and teeth, we pass by any particular notice of the rest.

It is conceded on all hands that the teeth have a birth, development, growth, maturity, decay, and death, as all the other organs, and that they likewise have blood-vessels and nerves as the rest, thus giving them both circulation and sensation. Mr. Goodsir tells us that the germs of the temporary teeth are seen as early as the seventh week after conception, and that about the fourteenth week provision is being made for the ten anterior permanent teeth. The child is now in the mother's womb, and the teeth are developed and grow from the mother's blood. After the child is born the teeth continue their development and growth from the mother's milk till the time of weaning, when it assumes an existence independent of the mother, and takes other food. Here, then, we observe three great periods in the formation of the teeth: the two first being absolutely dependent on the blood of the mother for the original soundness and good constitution of the teeth; while the latter period depends upon the after diet for the perfection of this soundness and its future preservation.

The infant, it is well known, has twenty teeth; the youth twenty-eight; the man thirty-two. The infant's number, we are told, is not completed till about the thirtieth or thirty-sixth month, and are not all shed till about the thirteenth or fourteenth year. Now both sets of teeth are under the especial care of the dentist; he is consulted daily about them, and it is his duty to watch and protect them in their infantile and youthful development and growth as the only security to the future man of a sound and healthy denture. And how, we respectfully ask, can any honest man do this, or even presume to attempt to do it, when he is most profoundly ignorant of the very first principles of the science upon which his practice is based. These principles teach that he should know the character of the blood supplying the teeth of the infant in the mother's womb; that he should know the character of the milk furnished by the mother after the child is born, and finally, that he should know the character of the diet, to be formed into blood, for the teeth of youth and manhood. For from these three sources are drawn all the materials required for the construction of the teeth during these three periods of their development and growth. And if these sources be corrupted, and thereby incapable of supplying a proper, healthy material, it is impossible that we can have formed sound and healthy teeth. A knowledge of the first source, the material blood, requires an acquaintance with the constitution and habits of the mother and the condition of her organs as to health and disease by which her blood was formed, and which knowledge is the only safe criterion to the dentist in advising the patient about her health, as well as forming a proper estimate of the condition of the teeth of her future offspring, provided she does not heed his advice. The second and third sources, differ from the first, by being required to be formed into blood before they are fit to supply the teeth with proper nourishment, and this requires the additional study of the infant's constitution, with all its tender relations and most delicate susceptibilities. Now all such knowledge as this includes no less than a knowledge of anatomy, physiology, pathology, and hygiene; in other words, a knowledge of all the organs in all their relations, both in health and disease. But such qualifications as these are disclaimed as unnecessary—we have no allusion to the scientific dentist—and brings us back to our original complaint, that no dentist without such knowledge is competent to discharge his duty to his patients.

Dentists are often consulted as to the proper time of weaning a child. This is a point connected with nutrition, and is of great practical importance to the future well-being of the little patient.

The last number of the Dental Journal contains a clinical lecture by M. Trousseau upon this subject. He says, "the teeth of infants come out in groups. The first group consists of two middle inferior incisors at about eight months; second group, two middle superior incisors, at about ten months; third group, two lateral superior incisors at about a year; fourth group, two lateral inferior incisors and the first four molars (six teeth in this group) at fourteen to sixteen months; fifth group, four cuspids, at eighteen months to two years; sixth group, four second and last molars, at thirty or thirty-six months."

The practical point here is, the period of repose between each group, and which M. T. insists is the only proper and safe time for weaning; and even then not too soon, as the delicate state of the child's bowels, with its strong disposition to cholera infantum, would certainly endanger life from this disease, if the mother's milk be withheld. M. T. prefers waiting for the interval after the fourth group, or until the child has twelve teeth, before you advise weaning, as the organs are now better adapted for digesting and assimilating the new food with which it is provided; and it is this adaptation of the food to the organs, a knowledge of which enables the dentist to give an intelligent reason for this advice, which is so anxiously sought in this trying period of the child's existence.

Our remarks upon the blood, in its relation to the teeth, having already extended so far, allows us but a brief space for the few additional points we had proposed to notice. This, however, is the less to be regretted, as the blood is the fundamental and starting point to the dentist in all his investigations into the various qualities of the teeth, a thorough knowledge of which in its relations, not with one but with every organ, forms the chief basis for estimating the amount of influence which the various organs have upon the teeth, and the teeth upon the different organs, so that by being able to judge properly of this reciprocal influence he is able to turn the whole to practical account, and by so doing, to attain to far greater skill and success in his dental operations.

The organs related with the teeth, it has already been stated in a general way, comprise those of the whole body; and we shall here simply and briefly enumerate those only which are most prominently related with the teeth, as for example, the *salivary glands*, which furnish the principal fluids to the mouth, and which, along with the mucus, bathe the teeth, can from the character and action of these fluids be the means of either their preservation or destruction.

The *stomach* is directly connected with the mouth and the teeth, by means of a continuous mucous membrane extending through the pharynx and œsophagus. The connection is equally close by function, as both are engaged in the process of digestion—the one at its beginning and the other near its completion.

The *intestines*, small and large, come next, and are related by this same continuous mucous membrane, having poured into them a variety of fluids from various quarters: 1st, from the countless little glandular bodies within the coats of the tube itself; and second, from the liver and pancreas furnishing the bile and pancreating fluids, through whose agency it is known that the chyle is formed. Now this chyle, sometimes known as the white blood, owes its proper constitution entirely to the healthy relation existing between the dental organs on the one hand, and the intestines, with their appendages, on the other hand.

The *lungs* are also connected with the teeth and mouth by the mucous membrane extending down the trachea and bronchi, through which the air is constantly passing and repassing, and thus reciprocally affecting either class of organs accordingly as the air may be charged with gases that are deleterious.

The *brain* is directly connected with the teeth through the fifth pair of nerves, and often throws the system into the most violent convulsions during dentition.

The *uterus* is related with the teeth, but precisely in what way, is rather difficult to say; possibly, through Mr. Paget's theory of complemental nutrition, or through the spinal marrow and fifth pair of nerves. But the fact of the relation itself is unquestioned. Delabarre relates the following case:

"I know a lady of thirty, who, ten years ago, was of a fine constitution; her teeth were of an excellent quality; her gums fresh, and the small arterial vessels of the lips of a beautiful color. A painful delivery occasioned great disturbance in the economy; the matrix remained diseased. Then, there was great paleness of the gums and lips, on which soon but a few red vessels were perceived; the saliva was very stringy. One day, without the advice of the physician, she applied leeches; and the blood was very pale and serous. The general debility was necessarily found to be increased. Soon the teeth felt the want of sanguification; they were spotted in different places; caries was developed, and all local endeavors to restrain it were for a long time ineffectual; they rotted below the enamel, occasioning dull pains, and the caries presented a large excavation; a part of the osseous centre of each tooth was softened, its calcareous phosphate having been absorbed. Four were in a short time destroyed; but, finally, nature, seconding the efforts of the physician, her health was restored."

Though we do not assent fully to some of the explanations given in this case, yet it suffices to show the closeness of the relation between the teeth and the uterus, and the necessity of the dentist being familiar with this relation in his operations; otherwise he might destroy teeth not at all diseased, but only sympathetically affected.

The *spinal marrow*, from the recent experiments of M. Brown Seguard, before the French Academy of Sciences, when injured, is shown "to produce such a change in the vitality of the trigeminus nerve, or in that part of the brain at which this nerve terminates, that the excitation of its ramifications upon the face causes convulsions. The right half of the spinal marrow exerts this influence upon the right trigeminus nerve or right side of the brain, while the left half of the cord exerts the same influence upon the left nerve or side of the brain."

The nerve here spoken of is the fifth pair, which, besides supplying the face, is freely distributed to the teeth of both upper and lower jaws by means of its superior and inferior maxillary branches, thus establishing the connection between the teeth and spinal marrow, and the reciprocal influence of each in its departure from health.

The last remark we shall offer is to very briefly notice a few of the constitutional and other disturbances which affect the teeth.

Schill, in his Semeiology of the Teeth, says that in scurvy, phthisis, and scrofula the teeth are elongated, arising, however, he adds, from "retraction of the gum." That in scurvy, and from the use of mercury, they are less firm.

In typhus fever the teeth are coated with a brown and dark sordes, while a white coat is produced from a variety of affections.

In chlorosis and anæmia, this same authority describes the gums as pale, which is confirmed by general experience. In hemorrhoidal discharges and dysmorrhea the teeth are a purple red; and in scurvy, diabetes, mellitus, and from the use of mercury, a dark red.

"The receding of the gums from the necks of the teeth, and the process of denudation," says Mr. Waite, "depend either on a peculiar state of the system, or some constitutional cause."

It is unnecessary to multiply further examples, as we trust enough has been already said to prove that the qualifications of the dentist to practice with the highest efficiency upon the teeth as his favorite specialty, are and should be as great as those required of the physician, who takes any other specialty, as the cye and the ear, for example, for his especial practice.

For it must be remembered by the dentist that the teeth, equally with the eye, the ear, or any other organ or set of organs, are members also of the same body; have relations in common with the rest of the body; mutually act and react with each and all the organs; are consequently influenced by all, and must therefore be studied in connection with all, for the greater skill and success in his own especial department.

